# Calcium Phosphate Loaded with Curcumin Prodrug and Selenium Is Bifunctional in Osteosarcoma Treatments

**DOI:** 10.3390/jfb15110327

**Published:** 2024-11-03

**Authors:** Mingjie Wang, Chunfeng Xu, Dong Xu, Chang Du, Yuelian Liu

**Affiliations:** 1 Academic Centre for Dentistry Amsterdam (ACTA), Department of Oral Cell Biology, Vrije Universiteit Amsterdam and University of Amsterdam, 1081 LA Amsterdam, The Netherlands; m.wang@acta.nl (M.W.); cfxu1987@outlook.com (C.X.); 2 Department of Biomaterials, School of Materials Science and Engineering, South China University of Technology, Guangzhou 510641, China; xudongac@163.com; 3 National Engineering Research Center for Tissue Restoration and Reconstruction, South China University of Technology, Guangzhou 510006, China; 4 Key Laboratory of Biomedical Materials and Engineering of the Ministry of Education, Innovation Center for Tissue Restoration and Reconstruction, South China University of Technology, Guangzhou 510006, China

**Keywords:** biomimetic calcium phosphate, selenium, curcumin, osteosarcoma, osteogenesis, ROS

## Abstract

Although SeO_3_^2−^ ions have been loaded onto calcium phosphate to treat a wide range of cancers, the quest to promote bone tissue regeneration is still ongoing. Curcumin (cur), an herbal extraction, can selectively inhibit tumor cells and promote osteogenesis. In this study, SeO_3_^2−^ ions were co-precipitated in biomimetic calcium phosphate (Se@BioCaP), and modified curcumin prodrug (mcur) was adsorbed on diverse Se@BioCaP surfaces (mcur-Se@BioCaP-Ads). Co-precipitation yielded Se@BioCaP with a significantly higher Se content and exhibited a tailorable micro-/nanostructure. The favorable pH-responsive release of Se and mcur from mcur-Se@BioCaP-Ads showed a synergistic anticancer efficiency in OS cells, enhancing OS cell inhibition more than a single dose of them, which might be associated with ROS production in OS cells. In addition, increased alkaline phosphatase activity and calcium nodule formation in MC3T3-E1 pre-osteoblasts were also verified. These results suggest this novel mcur-Se@BioCaP-Ads has promising and widespread potential in OS treatments.

## 1. Introduction

Osteosarcoma (OS) is a primary bone cancer that is mostly diagnosed in juveniles and the elderly population [1,2]. It is typically aggressive with a roughly 54% five-year survival rate. However, in patients suffering from pulmonary metastasis, the five-year survival drops to 19–30% [3,4]. Although some novel remedies have been applied in cancer treatments, surgery is still the mainstream treatment of OS. In addition, neoadjuvant and adjuvant chemotherapies mainly using methotrexate, doxorubicin, and cisplatin have also improved the five-year survival rate of OS [4,5,6]. Nonetheless, surgical resection and systemic applications of chemotherapeutics have some grievous complications, including severe bone defects, renal damage, impaired hearing, and cardiotoxicity.

The local delivery of anticancer agents is more popular than systemic medications in the clinic due to increased local concentrations and limited off-target effects. For targeted treatments, various materials, such as polymers, hydrogels, and liposomes, have been employed to deliver therapeutic agents. Given the native calcium phosphate (CaP)-rich environment and the demands for mechanical properties of implanted material in bone tissue, compared to other drug carriers, biomimetic CaPs (BioCaPs) are more promising for OS treatment, which is ascribed to their inorganic compositions that are identical to those of natural bone tissue and have a similar structure to hydroxyapatite in bone.

Various CaP biomaterials have been reported for drug delivery, mainly including hydroxyapatite (HA), tricalcium phosphate (β-TCP), and octacalcium phosphate (OCP). HA has a structure and inorganic composition identical to those of natural bone, and it is the first artificial CaP material used in bone formation. Owing to the abovementioned advantages, the application of HA was expanded to drug delivery to treat bone cancer [7,8]; however, the slow degradation rate of HA limits its clinical application. By contrast, β-TCP exhibits rapid dissolution in the body [9], but this may lead to the intense release of loaded drugs in a short period, diminishing the treatment effect and wasting the drug. OCP is considered the precursor of the biological HA formation of organic bone tissue. Compared to other CaP bone substitutes, OCP potentiates bone formation [10]. Most of these traditional CaP materials should be synthesized at a high temperature, needing sophisticated facilities. Alternatively, in this study, a novel CaP material that can be fabricated via a wet-biomimetic mineralization process at ambient temperature was investigated for drug delivery. This system consisted of three components: the β-TCP core, the inner amorphous CaP layer, and the outer OCP layer, leading to an ideal degradation rate and release of loaded drugs for bone formation and OS cell suppression.

To circumvent the disadvantages caused by chemotherapeutics used in current OS treatments, there are a variety of alternatives, such as selenium (Se) and curcumin (cur), that have been evaluated. Se, a trace element, is involved in numerous physiological and pathological events, including carcinogenesis, inflammation, immunomodulation, and antiviral activity [7,8]. It has been confirmed that Se has potent toxicity to diverse cancer cells, and comfortingly, this toxic effect is selective to cancer cells [11]. It is believed that Se eliminates cancer cells via its accumulation in cancer cells, which inevitably spurs the generation of intercellular reactive oxygen species (ROS) [11]. These excessive ROS irreversibly damage proteins, lipids, and DNA in cancer cells, initiating the apoptosis of affected cancer cells.

However, the anti-OS efficiency of Se alone is far inferior to chemotherapeutics used in the clinic. Thus, efforts to increase the anti-OS capacity of Se have been made by a combination strategy. Cur, a polyphenol extracted from *Curcuma longa*, has achieved increasing attention in OS treatment considering its tumorphilic toxicity and pro-osteogenesis [12,13]. However, cur is hydrophobic and has disappointing bioavailability in the human body. Therefore, for cur’s extended applications and improved treatment outcomes, polyethyleneglycol (PEG)-modified cur, i.e., modified soluble curcumin (mcur), was synthesized with profound water-solubility and bioavailability [10,14]. In this study, the combination of mcur and Se was expected for more potent OS cell suppression.

Se-loaded calcium phosphate bone substitutes have been reported to successfully inhibit OS cells [15,16]. On the other hand, a biomimetic CaP bone substitute that can sustainedly release bone morphogenetic protein-2 (BMP-2) has been reported [17]. Based on this, in the present research, Se-incorporated BioCa (Se@BioCaP) was fabricated for the potential sustained release of SeO_3_^2−^. Afterward, mcur was absorbed on the surface of Se@BioCaP (mcur-Se@BioCaP-Ads) for further OS cell suppression and bone formation. During the fabrication, various ratios of mcur, Se, and BioCaP were assayed for the optimal suppression effect and osteogenesis.

## 2. Materials and Methods

### 2.1. Materials

The 143B OS and MC3T3-E1 OB cell lines were obtained from the American Type Culture Collection (ATCC, Manassas, VA, USA). Mcur was provided by Professor Chang Du’s group, and the synthesis method is detailed in the previous literature [18]. Simulated body fluid (SBF) solution was purchased from Sigma-Aldrich (St. Louis, MO, USA). Fetal bovine serum (FBS), Dulbecco’s Modified Eagle Medium (DMEM), and trypsin with EDTA were purchased from Gibco BRL (Gaithersburg, MD, USA). The Alamar Blue solution and Reactive Oxygen Species Assay Kit were purchased from Solarbio (Shanghai, China). The alkaline phosphatase assay kit and Alizarin Red staining kit were obtained from Biyotime (Shanghai, China), and the β-TCP particles were from EPRUI (Shanghai, China).

### 2.2. Preparation of Mcur-Se@BioCaP-Ads

Se@BioCaP was prepared through a well-established wet biomimetic mineralization process [19]. Briefly, β-TCP as the core particles was immersed in a five-fold concentrated SBF solution and the pH was adjusted to 6 at the ambient temperature. Thereafter, the solution was stored at 37 °C for 24 h with gentle shaking. A thin layer of amorphous calcium phosphate will form on the surface of the core particles, serving as a substrate for crystalline layer deposition. A supersaturated calcium phosphate solution (containing 137 mM NaCl, 4 mM CaCl_2_·2H_2_O, and 2 mM Na_2_HPO_4_·2H_2_O) was prepared, and the final pH of this solution was 7.4 by being buffered with Tris buffer (72 mM). Na_2_SeO_3_ was also added to the solution for the final concentrations of 100, 200, 400, and 800 μg/mL, respectively. After 48 h, the products, coated with a crystalline calcium phosphate layer that incorporates Na_2_SeO_3_, were designated as Se@BioCaP100, Se@BioCaP200, Se@BioCaP400, and Se@BioCaP800, respectively, based on the Na_2_SeO_3_ concentrations. Mcur was adsorbed on the surface of Se@BioCaP at a 1:200 weight ratio through surface adsorption. Finally, mcur-Se@BioCaP-Ads granules were obtained via lyophilization.

### 2.3. Physiochemical Characterization of Se@BioCaP

The topography of the Se@BioCaP was observed using a scanning electron microscope (FE-SEM, Nova Nano SEM 430, Hillsboro, OR, USA). Energy dispersive spectroscopy (EDS) was applied to quantify the surface element distribution. The transmission electron microscopy (TEM) and selected area electron diffraction (SAED) pattern were recorded (Talos 120c, FEI, Thermo, Waltham, MA, USA) at 100 kV. The Se@BioCaP was placed on the copper sheets, and sputter-coated with a thin layer of platinum to enhance the conductivity and image quality. The crystal size of diverse Se@BioCaP was quantitatively determined (1.54j version, NIH, Bethesda, MD, USA) by measuring the average crystal size. The drug loading was quantified via direct measurement [20]. Mcur-Se@BioCaP-Ads particles were immersed in 1 M HCl for 10 min and centrifuged at 5000 rpm for 5 min. The supernatant was collated, inductively coupled plasma optical emission spectrometry (ICP-OES, iCAP 7000, Thermo Fisher Scientific, MA, USA) was used to determine the Se content, and the amount of mcur from degraded mcur-Se@BioCaP-Ads was measured through UV absorption spectrophotometry (SUNRISE, Thermo Fisher Scientific, MA, USA) at 405 nm. The release rate was measured by placing the mcur-Se@BioCaP-Ads in 1 mL PBS (pH 6.5 and 7.4, respectively) at 37 °C. PBS was collected and updated periodically. Samples were centrifuged at 5000 rpm for 5 min, and the supernatant was retrieved. Thereafter, the released Se was measured by ICP-OES, and mcur was quantified in the UV spectrophotometer at 405 nm.

### 2.4. In Vitro Cytoviability Assessments

The 143B OS and MC3T3-E1 OB cells (10,000/cm^2^) were seeded in 96-well plates and cultured in DMEM supplemented with 10% fetal bovine serum and 1% Penicillin-Streptomycin-Fungizone (Gibco, New York City, NY, USA) in an incubator at 37 °C under an atmosphere of 5% CO_2_. OS and OB cells were treated with BioCaP, Se@BioCaP, and mcur-Se@BioCaP-Ads for 1, 2, and 4 days, and the culture medium was replenished every 2 days. After the treatment, the medium was replaced by 10% Alamar Blue stock solution and cultured for 4 h at 37 °C in the dark; the absorption intensity was recorded by a microplate reader at a wavelength of 560/590 nm.

### 2.5. ROS Detection

143B cells were seeded in 96-well plates at a density of 10^4^/cm^2^. After the attachment, these cells were treated with completed medium or relative agents for 24 h, and the final concentration of these agents was 2.5 mg/mL. Thereafter, the medium was replaced with 5 µM 2′,7′-dichlorodihydrofluorescein diacetate solution diluted using a serum-free medium. 143B cells were stored at 37 °C in the dark for 30 min. Finally, these cells were rinsed, and the fluorescence of ROS was detected using a fluorescence microscope (Leica, Germany) wavelength at λ_ex_= 488 nm, λ_em_ = 520 nm, and ROS were quantitatively determined using ImageJ (1.54j version, NIH, MD, USA) through measuring the average fluorescence intensity.

### 2.6. Osteogenesis Assay

The osteogenic differentiation of MC3T3-E1 cells was determined by ALP activity and extracellular matrix (ECM) mineralization with ALP and Alizarin Red staining, respectively. The ALP activity was evaluated using an alkaline phosphatase assay kit on day 7. Briefly, MC3T3-E1 cells were seeded in a 96-well plate (5000 cells/well) and cultured overnight in complete growth medium to 70% confluence. Cells were then induced with osteogenic differentiation medium (consisting of 10 mM of β-glycerophosphate, 100 nM dexamethasone, and 0.2 mM ascorbic acid) with or without mcur-Se@BioCaP-Ads. The medium changed every 2–3 days. ALP (at day 7) and ARS (at day 14) were used to assess the effects of mcur-Se@BioCaP-Ads on the osteogenic differentiation of MC3T3-E1 cells. Alizarin Red S staining was used to observe calcium phosphate nodules. Briefly, cells were fixed in 4% formaldehyde and washed with deionized water 3 times. Then, 33 µL of 40 mM ARS (Sigma-Aldrich, St. Louis, MO, USA) in PBS for 20 min at 37 °C. The ARS was discarded, the sample was washed with deionized water until the supernatant was clear, then the ARS was extracted using cetylpyridinium chloride solution for 2 h at room temperature and quantified using the UV spectrophotometer at 405 nm.

## 3. Statistical Analysis

Statistical analysis was performed with SPSS 24.0 (IBM, Armonk, NY, USA). Data were presented as the means ± standard deviations. Differences between groups were analyzed by independent *t*-test and One-way Analysis of Variance (ANOVA) with Bonferroni’s post hoc test. A value of *p* < 0.05 was considered statistically significant.

## 4. Results

### 4.1. Characterizations of Se@BioCaP

**Figure 1:**
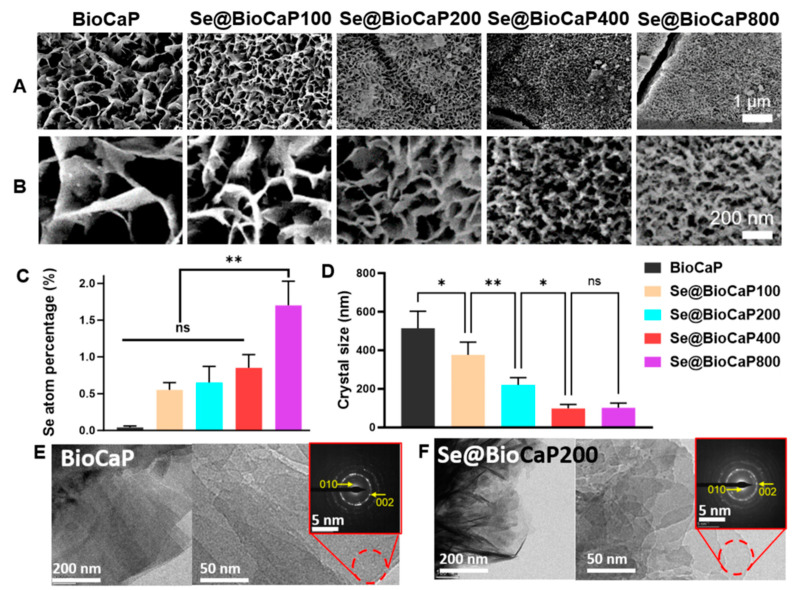
Characterizations of BioCaP and Se@BioCaP. (**A**) BioCaP and Se@BioCaP crystals synthesized with different doses of Na_2_SeO_3_ in mineralization solution; (**B**) BioCaP and Se@BioCaP crystal morphology of diverse Se@BioCaP crystals; (**C**) crystal atom percentage of Se in each Se@BioCaP sample; (**D**) crystal size of each Se@BioCaP sample. (**E**) TEM image and SAED pattern of BioCaP. (**F**) TEM image and SAED pattern of Se@BioCaP200 sample. Statistical difference: * p < 0.05, ** p < 0.01, ns *p* > 0.05.

In this study, BioCaP and a series of Se@BioCaP samples through biomimetic mineralization were fabricated. BioCaP showed flake-like calcium phosphate crystals, precipitated uniformly on the scaffold surfaces (Figure 1A). With the increased doses of Na_2_SeO_3_ in the mineralization solution from 100 to 800 µg/mL, the crystal size and roughness of the particle decreased instead (Figure 1A, B). This altered geometrical morphology suggested that Na_2_SeO_3_ was successfully incorporated into BioCaP. The subsequent analysis revealed differences in the micro-/nanostructure and elements of the BioCaP coatings were influenced by the concentration of Na_2_SeO_3_ in the mineralization solution.

Se@BioCaP exhibited significantly higher surface crystal densities compared to pure BioCaP (Figure 1A). Figure 1B showed the various crystal morphologies in diverse Se@BioCaP samples, showing that the introduction of Na_2_SeO_3_ led to a morphological shift from a straight flake-like structure to a curved one in the Se@BioCaP-100 and Se@BioCaP-200 (Figure 1B). Moreover, the crystal size of the Se@BioCaP-400 and Se@BioCaP-800 groups significantly decreased to 98 ± 21 nm and 102 ± 24 nm, respectively (Figure 1B,D). The size and shape of the crystals in the Se@BioCaP series were found to be correlated with the Na_2_SeO_3_ concentration and Se atom percentage (Figure 1C).

The data from the analysis of the BioCaP and Se@BioCaP-200 samples obtained by the TEM are presented in Figure 1E,F. The Se@BioCaP-200 crystal grew into a plate-like shape similar to that of the BioCaP crystals. The red circles indicate the diffraction area in the magnified SAED pattern, depicting the planes corresponding to 010 and 002, indicating the possibility of octacalcium phosphate being present [21].

The surface Se atom percentage (%Se) dose-dependently increased with the amount of Na_2_SeO_3_ used in the mineralization solution, as evident from the EDS mapping of the Se content (Figure S1). Also, the surface atom ratio of phosphorus (P) and calcium (Ca) significantly increased to 15.42 ± 0.9 and 24.75 ± 0.5 as 100 µg/mL Na_2_SeO_3_ was introduced into the BioCaP mineralization solution (Supplementary Table S1), while this dose-dependently decreased as the Se content increased. In addition, only Se@BioCaP100 showed a significantly higher Ca/P and Ca/(P + Se) ratio than BioCaP (Supplementary Table S1).

### 4.2. Drug Loading and Release Kinetics of Mcur-Se@BioCaP-Ads

**Figure 2:**
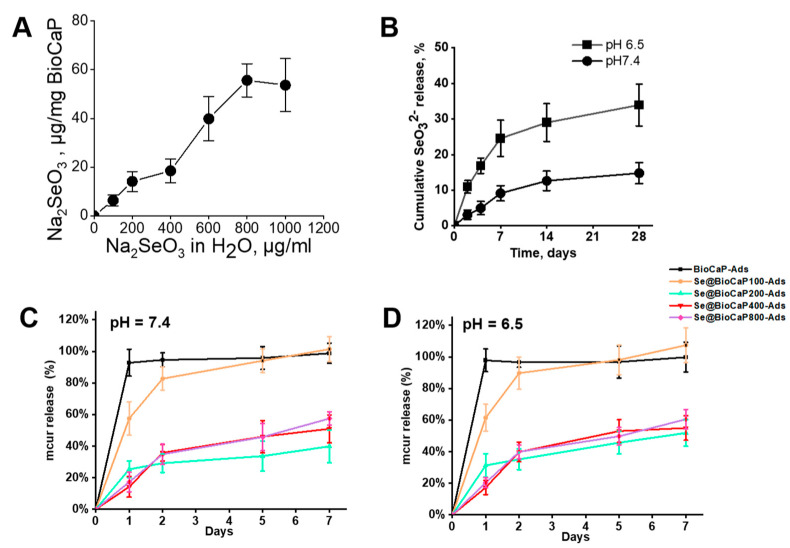
Drug loading and release rates of Se@BioCaP. (**A**) Na_2_SeO_3_ content in diverse Se@BioCaP-Ads; (**B**) Na_2_SeO_3_ release curves in acidic and neutral environments; (**C**,**D**) release curves of mcur absorbed on the surfaces of BioCaP or various Se@BioCaP carriers in pH 7.4 and pH 6.5.

The surface Se% distribution was increased from 0.55 ± 0.3% to 1.7 ± 0.3% of the Se@BioCaP (Table 1). The content of Na_2_SeO_3_ loaded onto BioCaP increased from 6.3 to a maximum of 56 µg/mg when the concentration of Na_2_SeO_3_ solution rose from 100 to 800 µg/mL (Table 1). Na_2_SeO_3_ almost reached the maximum loading capacity when the concentration was about 800 µg/mL (Figure 2A). The drug release rate is a vital parameter for local drug delivery in bone tumor treatments and bone tissue regeneration. Therefore, the Na_2_SeO_3_ release rates of mcur-Se@BioCaP-Ads were investigated under healthy and bone tumor conditions (pH 7.4 and pH 6.5, respectively) [22]. As shown in Figure 2B, the release of Na_2_SeO_3_ in the pH 6.5 environment was much more rapid than that under pH 7.4. In vitro, in pH 6.5, after 28 days, more than 30% of the loaded Na_2_SeO_3_ was released. However, only around 10% was detected in pH 7.4.

A rapid mcur release was also observed in this study. After only 24 h, mcur was almost totally released from the surface of the pure BioCaP carrier, regardless of whether it was under pH 7.4 (Figure 2C) or pH 6.5 (Figure 2D). Among the mcur-Se@BioCaP-Ads groups, mcur-Se@BioCaP200-Ads, Se@BioCaP400-Ads, and Se@BioCaP800-Ads showed similar release rates and displayed a slower release rate than that of BioCaP as well as the Se@BioCaP100-Ads group. However, in the Se@BioCaP200-Ads group, only approximately 40% of the mcur was released over seven days.

### 4.3. Cytoviability Assessments

**Figure 3:**
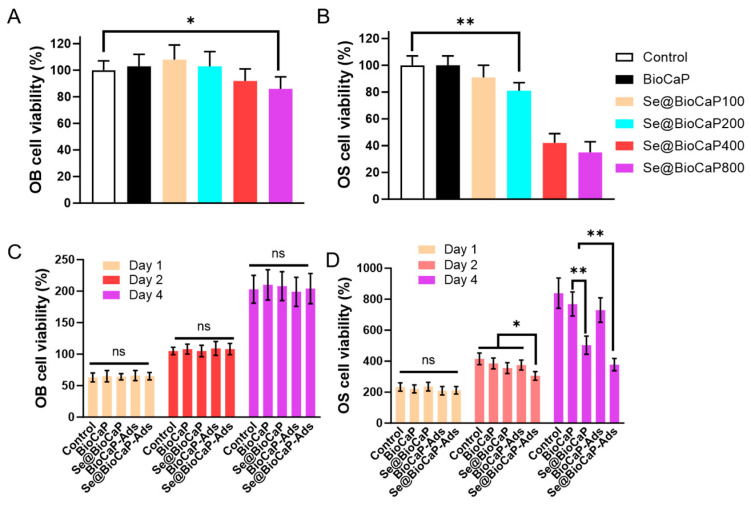
Viability of osteoblasts and OS cells after being treated by different materials. (**A**) Viability of pre-osteoblasts (MC3T3-E1) treated with BioCaP and diverse Se@BioCaPs; (**B**) viability of OS cells treated with BioCaP and diverse Se@BioCaPs; (**C**) viability of pre-osteoblasts (MC3T3-E1) treated with mcur-Se@BioCaP200-Ads at day 1, 2, and 4. (**D**) Viability of OS cells treated with mcur-SeBioCaP200-Ads at day 1, 2, and 4. Statistical difference: * p < 0.05, ** p < 0.01, ns *p* > 0.05.

Based on ISO-10993, an implanted medical device should not decrease the cytoviability of normal cells to below 70% [23]. Although Na_2_SeO_3_ and cur are tumor-selective, an overly high concentration of each of them is still toxic to healthy cells, including osteoblasts. Therefore, the cytotoxicity of Se@BioCaP to MC3T3-E1 cells was detected first (Figure 3A). As shown in Figure 3A, except for the Se@BioCaP800 group (*p* < 0.05), other Se@BioCaP samples showed subtle cytotoxicity. Regarding OS inhibition, BioCaP and Se@BioCaP100 did not inhibit OS cell viability, while Se@BioCaP200, Se@BioCaP400, and Se@BioCaP800 significantly decreased the cell viability to 81 ± 6%, 42 ± 7%, and 35 ± 8% (Figure 3B) (*p* < 0.05). Based on these results and due to the similar crystal morphology and drug release rate of Se@BioCaP 200 with BioCaP, Se@BioCaP200 was used in the following study.

To evaluate the effect of mcur-Se@BioCaP200-Ads on the cytoviability of MC3T3-E1 cells and 143B cells, these two types of cells were treated with mcur-Se@BioCaP200-Ads. After a 4-day culture, mcur-Se@BioCaP200-Ads was not toxic to the OB cells (Figure 3C). On the other hand, on day 2, only mcur-Se@BioCaP200-Ads decreased the viability of the 143B cells (*p* < 0.05). Moreover, on day 4, mcur-Se@BioCaP200-Ads demonstrated the most potent inhibition of the 143B cells (*p* < 0.01).

### 4.4. Accumulation of ROS

**Figure 4:**
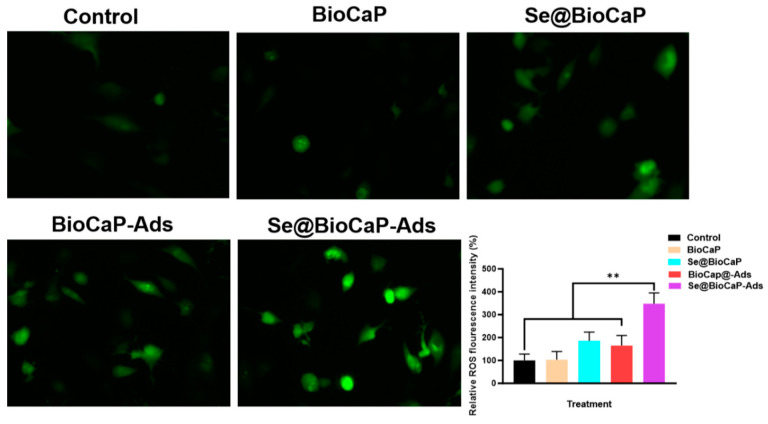
ROS staining of treated 143B cells in each group. Statistical difference: ** p < 0.01.

Following the viability assessments, the ROS level of the treated 143B cells was detected. As showed in Figure 4, mcur-Se@BioCaP200-Ads drastically induced ROS overproduction in the 143B cells with a more intense fluorescence signal of the ROS dye (*p <* 0.01).

### 4.5. Osteogenesis

**Figure 5:**
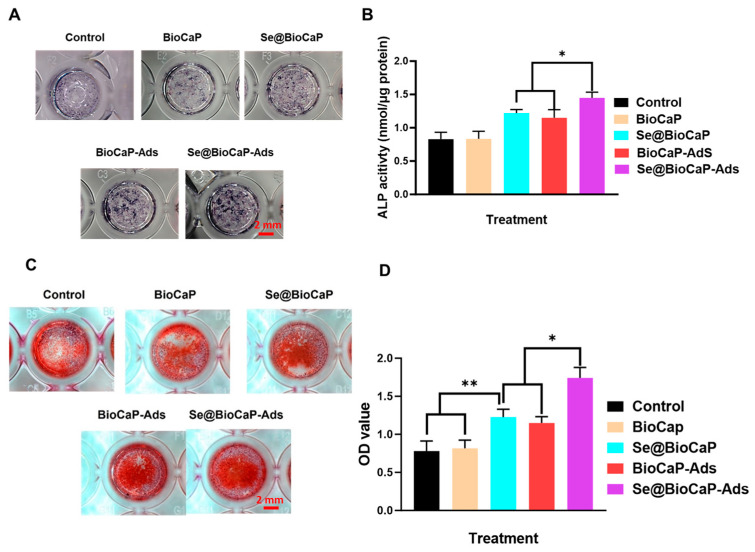
Osteogenesis in each group. (**A**) ALP activity staining in each group on day 7, bar = 2 mm; (**B**) ALP activity of OB cells, *n* = 3; (**C**) ARS staining on day 14, bar = 2 mm. (**D**) Quantitative analysis of ARS staining on day 14, *n* = 3. Statistical difference: * p < 0.05, ** p < 0.01.

The pro-osteogenesis of mcur-Se@BioCaP200-Ads was also evaluated in this study via evaluating the ALP activity and calcific nodules. Following the osteogenic induction of the MC3T3-E1 pre-osteoblasts, ALP (Figure 5A) and calcium nodule (Figure 5C) formation were found in the Se@BioCaP200, BioCaP-Ads, and mcur-Se@BioCaP200-Ads groups. Mcur-Se@BioCaP200-Ads showed a great increase compared with the control group and BioCaP group (Figure 5B,D) (*p* < 0.05). Further, mcur-Se@BioCaP200-Ads significantly increased the ALP activity to 1.45 ± 0.08 nmol/µg protein, which is significantly higher than that of the Se@BioCaP200 and BioCaP-Ads groups (Figure 5B) (*p* < 0.05). Consistent with the ALP data, the Alizarin Red staining results also indicated that mcur-Se@BioCaP200-Ads induced the most calcific nodules in two weeks.

## 5. Discussion

Progress in OS treatments has remained at a standstill over recent decades. To date, the strategy, surgery with combined MAP (methotrexate, doxorubicin, cisplatin) chemotherapy, is still the primary remedy for OS treatments. Although this strategy improves the five-year survival rate of OS patients, compared with surgery alone, two grievous shortcomings, i.e., severe surgery-associated bone defects and cytotoxicity to healthy cells, that arise from chemotherapeutics, overshadow the clinical outcomes of current OS treatments.

To resolve these issues, some multifunctional agents were investigated to cure OS. These agents are expected to be tumor-targeted toxic and pro-osteogenic. Among these candidates, Se receives increasing attention as it orchestrates a series of physiological events in the body, such as carcinogenesis, inflammation, immunomodulation, and antiviral activity [24]. Of note, it has been well documented that Se is specifically toxic to cancer cells [25]. Consequently, this trace element is involved in our study. However, Se species, such as selenomethionine, selenocysteine, selenium-methyl selenocysteine, SeO_3_^2−^, and SeO_4_^2^, play disparate roles in various cancer and OS cell models [26,27,28]. For example, Huang et al. reported that Se-methyl selenocysteine inhibited MG-63 cells’ proliferation but stimulated the growth of U2OS cells instead [29]. In our research, only one OS cell line, 143B, was used to evaluate the OS inhibition effect of mcur-Se@BioCaP-Ads. Therefore, there is concern that our data did not reflect the authigenic effect of mcur-Se@BioCaP-Ads on OS cells in the body, which may misguide further in vivo assessments, even clinical trials. To diminish this risk, more distinct human OS cell lines should be recruited to confirm the consistent anti-OS ability of mcur-Se@BioCaP-Ads in vitro before subsequent in vivo studies and potential clinical trials in the future.

Although inorganic Se species, containing SeO_3_^2−^, SeO_4_^2−^, and SeO_2_, have remarkable biosafety profiles for healthy normal cells, a high concentration of them is also fatal for normal healthy cells [29]. Hence, the sustained release of SeO_3_^2−^ from the CaP carrier should be achieved to eschew the local overconcentration of SeO_3_^2−^. In previous studies, This BioCaP drug delivery system has been tested for drug release evaluation. Various bioagents, from macro- to micro-molecules, including bovine serum albumin, BMP-2, and mcur were successfully incorporated into these CaP bone substitutes, respectively, and their sustained release was detected [17,30]. Na_2_SeO_3_, ~173 g/mol, was successfully encapsulated into this CaP material, and its sustained release was observed in the present study, even in an acidic environment that promotes the rapid degradation of CaP materials. However, the dissolution of CaP materials in vitro is mainly ascribed to hydrolysis, whereas, in vivo, due to the foreign body reaction, the implanted CaP bone substitutes will also be degraded by multinucleated giant cells and osteoclasts, leading to more rapid degradation than hydrolysis in vitro. Thus, an appropriate OS animal model should be used to analyze the authigenic degradation rate of Se@CaP-Ads in vivo, thereby providing solid evidence of the sustained release of SeO_3_^2−^ in vivo.

The structures of materials can also affect the release of loaded drugs. In this research, Na_2_SeO_3_ at appropriate concentrations was successfully incorporated into BioCaP crystal and modulated the crystal growth. Se@BioCaP showed a curved crystal morphology and significantly increased P atom ratio, which could be interpreted as the incorporation of Na_2_SeO_3_ increasing the crystal surface availability [11]. However, a decreased crystal size and destroyed crystal lattice (Figure 2B) were observed in the high-concentration SeO_3_^2−^ groups, i.e., the Se@BioCaP400 and Se@BioCaP800 groups. We did our best to explain the potential mechanism. SeO_3_^2−^ has a pyramidal oxyanion structure, which is the most dominant ionic substitute for phosphates in biogenic apatite when compared to phosphate tetrahedrons [1]. High-dose SeO_3_^2−^ in BioCaP mineralization solution replaced the PO_4_^3−^ and destroyed the BioCaP crystal shape. Therefore, the crystal size and phosphorus atom percentage were both decreased. In this study, since Se@BioCaP400 and Se@BioCaP800 showed toxic effects on MC3TE-E1 cells, these two groups were not involved in the following assessments, including Se release rate evaluations. Hence, the exact release rates of SeO_3_^2−^ from these groups are still unknown. Regarding whether the high-dose SeO_3_^2−^ is toxic to healthy cells, it may be suspected that the toxicity of Se@BioCaP400 and Se@BioCaP800 to MC3TE-E1 cells can be attributed to the rapid release rates of SeO_3_^2−^ as a result of altered crystal structures. However, due to the technical difficulties of peeling off enough pure Se@BioCaP coating, X-ray diffraction (XRD) was not performed to determine the exact crystal type to analyze its influence on SeO_3_^2−^ release.

Although the sustained release of SeO_3_^2−^ can restrain the potential damage to normal cells attributed to an overtly high concentration of SeO_3_^2−^, instead, an insufficient concentration of SeO_3_^2−^ may be incapable of eradicating OS cells. Given this concern, mcur was absorbed on the surface of Se@BioCaP to improve the OS inhibition efficiency. As tumorphilic as SeO_3_^2−^ is, cur is also selective for cancer cells and kills OS cells via an enhanced ROS level, one of the mechanisms of its anticancer capacity. Accordingly, cur can suppress OS via autophagy, ferroptosis, etc. [1,2]. Hereby, we hope mcur and SeO_3_^2−^ have synergistic or additive effects to ensure improved OS inhibition. Consequently, after the fabrication of Se@BioCaP, we further modified it with mcur absorption on its surface. However, based on the viability of 143B cells, the mcur absorbent did not endow a 143B cell suppression ability to BioCaP but reinforced Se@BioCaP in 143B cell inhibition. This result testified that SeO_3_^2−^ and mcur have a synergistic/additive effect on inhibiting 143B cells, but the potential mechanism has not been uncovered in this research. On the other hand, the enhanced bone formation has been validated in the Se@BioCaP, BioCaP-Ads, and mcur-Se@BioCaP-Ads groups. Based on this, the effect of SeO_3_^2−^ on bone formation was illustrated, which is consistent with previous reports [31,32], and mcur also showed a synergistic/additive effect on bone formation with SeO_3_^2−^. Due to the rapid release of mcur from the surface of mcur-Se@BioCaP-Ads, encapsulating mcur into BioCaP with Na_2_SeO_3_ simultaneously may have more positive outcomes, while regarding a prudent consideration, we did not encapsulate mcur into CaP as we are unaware if the slow release rate of mcur or the interaction between mcur and Na_2_SeO_3_ will attenuate the treatment effect. In light with the data from this study, the synergistic effect of these two drugs was identified. Thus, we attempt to synthesize an mcur- Na_2_SeO_3_ co-incorporated CaP bone substitute for OS treatment with a more elevated efficiency than that of mcur-Se@BioCaP-Ads.

Another advantage of this CaP delivery system is the enhanced bone defect rehabilitation, which has been not given more attention in clinical conditions. In comparison to other delivery systems made of hydrogels, polymers, and liposomes, the CaP biomaterial has a more favorable biocompatibility regarding its inorganic composition that is identical to that of natural bone tissue and that has a similar structure to that of natural hydroxyapatite. Thus, from a safety-conscious point of view, CaP biomaterial is more safe than other biomaterials in a bone cancer setting. However, the discrepancies among various CaP materials cannot be ignored. It has been suggested that the CaP biomaterials synthesized under conditions similar to those of biomineralization processes in the body can have a direct inducing effect on bone formation [33]. The CaP carrier used in our study was fabricated via a precipitation process at 37 °C under mild pH conditions. Regarding these, the pro-osteogenesis effect of this CaP material may surpass that of pure HA and β-TCP. In this study, the mechanical properties of this CaP biomaterial were not evaluated and compared with other CaP bone substitutes, weakening our comprehensive understanding of it and the exploration of its potential applications in 3D printing.

## 6. Conclusions

The results of this study demonstrated that this SeO_3_^2−^ and curcumin functionalized CaP biomaterial has low toxicity to MC3T3-E1 cells, while it is selectively toxic to 143B cells. Meanwhile, the synergetic effect of loaded SeO_3_^2−^ and curcumin advanced osteogenesis and osteosarcoma cell inhibition in vitro.

## Figures and Tables

**Table 1:** Na_2_SeO_3_ contents in Se@BioCaP measured by ICP-OES and EDS (8 samples per group).

Characterization Equipment	Na_2_SeO_3_ in Mineralization Solution, µg/mL	100	200	400	800
ICP-OES	Na_2_SeO_3,_ µg/mg BioCaP	6.33	14.11	15.46	55.64
	SD	1.67	2.64	2.73	5.34
EDS	%Se	0.55	0.65	0.85	1.7
	SD	0.3	0.2	0.2	0.3

## Data Availability

The original contributions presented in the study are included in the article/supplementary material, further inquiries can be directed to the corresponding authors.

## Supplementary Materials

The following supporting information can be downloaded at: https://www.mdpi.com/article/10.3390/jfb15110327/s1, Figure S1: Surface Se atom distribution measured through SEM-EDS mapping; Table S1: Surface atomic percentages. All percentages are reported as mean value ± SD, *n* = 3.
