# Supplementary material for: Calcium Phosphate Loaded with Curcumin Prodrug and Selenium Is Bifunctional in Osteosarcoma Treatments
Source: J Funct Biomater. 2024 Nov 3;15(11):327. doi: 10.3390/jfb15110327 (PMC11594945; doi:10.3390/jfb15110327)
Supplement: Supplementary file 1 [file jfb-15-00327-s001.zip › jfb-3241872-supplementary.pdf]

Supplementary Materials

Calcium Phosphate Loaded with Curcumin Prodrug and Selenium Is Bifunctional in Osteosarcoma Treatments

Mingjie Wang [1,†], Chunfeng Xu [1,†], Dong Xu [2,3,4], Chang Du [2,3,4,*] and Yuelian Liu [1,*]

[1] Academic Centre for Dentistry Amsterdam (ACTA), Department of Oral Cell Biology, Vrije Universiteit Amsterdam and University of Amsterdam, 1081 LA Amsterdam, The Netherlands; m.wang@acta.nl (M.W.); cfxu1987@outlook.com (C.X.)
[2] Department of Biomaterials, School of Materials Science and Engineering, South China University of Technology, Guangzhou 510641, China; xudongac@163.com
[3] National Engineering Research Center for Tissue Restoration and Reconstruction, South China University of Technology, Guangzhou 510006, China
[4] Key Laboratory of Biomedical Materials and Engineering of the Ministry of Education, Innovation Center for Tissue Restoration and Reconstruction, South China University of Technology, Guangzhou 510006, China
***** Correspondence: duchang@scut.edu.cn (C.D.); y.liu@acta.nl (Y.L.); Tel.: +31-205-980-626 (Y.L.)
† These authors contributed equally to this work.

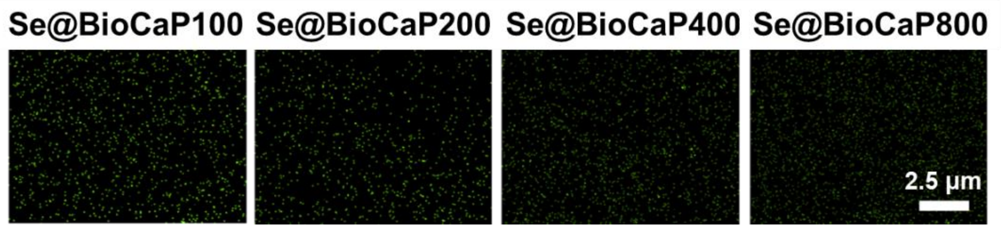

Figure S1. Surface Se atom distribution measured through SEM-EDS mapping.

Table S1. Surface atomic percentages. All percentages are reported as mean value ± SD, n=3.

Sample	%Se	%O	%P	%Ca	Ca/(P+Se)	Ca/P
BioCaP	-	59.86 ± 1.8	12.16 ± 0.8	17.67 ± 0.8	1.45 ± 0.05	1.45 ± 0.06
Se@BioCaP100	0.55 ± 0.3	47.43 ± 1.2 *	15.42 ± 0.9 *	24.75 ± 0.5 *	1.55 ± 0.03 *	1.61 ± 0.02 *
Se@BioCaP200	0.65 ± 0.2	49.23 ± 1.1 *	14.63 ± 0.6 *	22.38 ± 0.8 *	1.46 ± 0.06 ^{ns}	1.53 ± 0.05 ^{ns}
Se@BioCaP400	0.85 ± 0.2	50.58 ± 0.8 *	12.4 ± 0.7 ^{ns}	18.88 ± 1.1 ^{ns}	1.42 ± 0.02 ^{ns}	1.52 ± 0.03 ^{ns}
Se@BioCaP800	1.70 ± 0.3	56.39 ± 1 *	10.25 ± 0.8 *	16.02 ± 1.1 *	1.34 ± 0.07 ^{ns}	1.56 ± 0.06 ^{ns}

Statistical difference * $p < 0.05$, ^{ns} $p > 0.05$.
